# Occurrence of OXA-48 Carbapenemase and Other β-Lactamase Genes in ESBL-Producing Multidrug Resistant *Escherichia coli* from Dogs and Cats in the United States, 2009–2013

**DOI:** 10.3389/fmicb.2016.01057

**Published:** 2016-07-11

**Authors:** Xiaoqiang Liu, Kamoltip Thungrat, Dawn M. Boothe

**Affiliations:** ^1^Department of Basic Veterinary Medicine, College of Veterinary Medicine, Northwest A&F UniversityYangling, China; ^2^Department of Anatomy, Physiology and Pharmacology, College of Veterinary Medicine, Auburn UniversityAuburn, AL, USA

**Keywords:** *Escherichia coli*, ESBL, OXA-48 carbapenemase, multidrug resistance, companion animals

## Abstract

**Objective:** The aim of this study was to explore the occurrence and molecular characterization of extended-spectrum β-lactamases (ESBL), plasmid-mediated AmpC β-lactamase (pAmpC) and carbapenemases among ESBL-producing multidrug resistant (MDR) *Escherichia coli* from dogs and cats in the United States.

**Methods:** Of 2443 *E*.coli isolated from dogs and cats collected between August 2009 and January 2013, 68 isolates were confirmed as ESBL-producing MDR ones. PCR and sequencing were performed to identify β-lactamases and plasmid-mediated quinolone resistance (PMQR) genes, and shed light on the virulence gene profiles, phylogenetic groups and ST types.

**Results:** Phylogenic group D and B2 accounted for 69.1% of the isolates. 50 (73.5%) isolates carried CTX-M ESBL gene, and the most predominant specific CTX-M subtype identified was *bla*_CTX−M−15_ (*n* = 33), followed by *bla*_CTX−M−1_ (*n* = 32), *bla*_CTX−M−123_ (*n* = 27), *bla*_CTX−M−9_ (*n* = 19) and *bla*_CTX−M−14_ (*n* = 19), and *bla*_CTX−M−123_ was firstly reported in *E. coli* isolates in the United States alone or in association. Other β-lactamase genes *bla*_TEM_, *bla*_SHV_, *bla*_OXA−48_, and *bla*_CMY−2_ were detected in 41.2, 29.4, 19.1, and 17.6% of 68 ESBL-producing MDR isolates, respectively. The *bla*_TEM_ and *bla*_SHV_ genes were classfied as ESBLs with the exception of the *bla*_TEM−1_ gene. Additionally, 42.6% (29/68) of isolates co-expressed *bla*_CTX−M−15_ and PMQR gene *aac(6*′*)-Ib-c*. The overall occurrence of virulence genes ranged from 11.8 (*ireA*) to 88.2% (*malX*), and most of virulence genes were less frequent among CTX-M-producing isolates than non-CTX-M isolates with the exception of *malX* and *iutA*. The 68 isolates analyzed were assigned to 31 STs with six being novel. Three pandemic clonal lineages ST131 (*n* = 10), ST648 (*n* = 9), and ST405 (*n* = 9) accounted for more than 41% of the investigated isolates, and ST648 and ST405 of phylogenetic D were firstly reported in *E. coli* from dogs and cats in the United States.

**Conclusion:** *bla*_CTX−M−123_ of ESBLs and carbapenemase *bla*_OXA−48_ were firstly reported in ESBL-producing MDR *E*.coli from dogs and cats in the United States, and ST131, ST648, and ST405 were the predominant clonal groups.

## Introduction

Extraintestinal pathogenic strains of *Escherichia coli* (ExPEC) are the most important dogs and cats bacterial pathogens associated with extraintestinal infections (Beutin, 1999). However, extended-spectrum β-lactamase (ESBL)-producing ExPEC are isolated worldwide with increasing frequency from human and animal clinical isolates (Pitout, 2012). The occurrence of β-lactamases, including ESBLs, plasmid-mediated AmpC β-lactamases (pAmpC) and carbapenemases among *E. coli* pose serious challenges to the use of penicillins, extended-spectrum cephalosporins (3rd and 4th generation cephalosporins), monobactams, and carbapenems (Karisik et al., 2008; Geser et al., 2012). Furthermore, ESBL-producing isolates are often cross-resistant to fluoroquinolones and other antimicrobial agents, thus expressed multidrug resistance (MDR). This combination of properties can significantly affect the course and outcomes of infections. β-lactamase genes commonly located on mobile genetic elements, such as plasmids, transposons, or integrons, and the resistance plasmids can easily be transferred between bacterial isolates by conjugation mechanism. Accordingly, transmission of β-lactamase genes between companion animals and owner has become a subject of active discussion as companion animals could be potential sources of ESBL-producing *E. coli* isolates causing community-acquired infections (Schmiedel et al., 2014).

Although the ESBLs, pAmpCs and carbapenemases in *E. coli* isolates from humans and animals have been characterized in various studies around the world, knowledge about the β-lactamases and population structure in MDR *E. coli* isolates from companion animals in the United States is limited. Prior to the current study only two studies have described the occurrence and the diversity of ESBLs in *E. coli* from dogs and cats in the United States (O'Keefe et al., 2010; Shaheen et al., 2011), and the isolates were collected from September 2004 to December 2007, and May 2008 to May 2009, respectively. However, the β-lactamases, particularly CTX-M-type ESBLs, are characterized by ongoing and complex evolution. Currently, greater than 150 variants have been identified, and several chimeras, e.g., *bla*_CTX−M−64_ and *bla*_CTX−M−123_ have been reported since 2009 (He et al., 2013). Moreover, several novel β-lactamases, e.g., *bla*_KPC_, *bla*_NDM−1_, and *bla*_OXA−48_ are emerging worldwide in *E. coli* isolated from humans or animals.

The aim of the present study was to (i) investigate the occurrence and molecular characterization of ESBL-producing MDR *E. coli* recovered from clinical cases of infection in dogs and cats in the United States, over a period of time ranging from August 2009 to January 2013, and (ii) characterize the association between β-lactamases, phylogenetic groups, virulence genes and the ST types.

## Materials and methods

### Bacterial isolates

Between August 2009 and January 2013, a total of 2443 *E. coli* isolates from urine, wound, ear, genital tract, anal sac, nasal structure, and soft tissue samples of dogs and cats with presumed naturally-occurring infection in six geographical regions of the United States: West (California), South (North Carolina), Central (Missouri), Midwest (Ohio and Illinois), and Southeast (Alabama), Northeast (Massachusetts) were received from a nationally recognized veterinary diagnostic laboratory. Isolates were reconfirmed to be *E. coli* upon receipt by the Clinical Pharmacology Laboratory (CPL) at Auburn University based on reculture overnight on CHROMagar Orientation (BD Diagnostics, Franklin Lakes, NJ) at 37°C, and then the isolates were harvested and stored in tryptic soy broth containing 30% glycerol at −80°C until studied.

### Susceptibility testing and initial ESBL identification

Antimicrobial susceptibility testing was performed for all 2443 isolates using 96 well custom microdilution susceptibility plates according to the manufacturer's protocol (Trek Diagnostic Systems, Inc., Cleveland, OH). Susceptibility testing was performed using 16 antimicrobials representing six antimicrobial classes and classified into 12 antimicrobial categories: penicillins: ampicillin; penicillins + β-lactam inhibitor: amoxicillin-clavulanic acid; anti-pseudomonal + β-lactam inhibitor: ticarcillin-clavulanic acid; non-extended spectrum cephalosporins (1st generation cephalosporins): cephalothin; extended-spectrum cephalosporins (3rd and 4th generation cephalosporins): cefotaxime, ceftazidime, and cefpodoxime; cephamycins: cefoxitin; carbapenems: meropenem; tetracyclines: doxycycline; phenicols: chloramphenicol; fluoroquinolones: enrofloxacin and ciprofloxacin; aminoglycosides: gentamicin and amikacin; and folate pathway inhibitor: sulfamethoxazole-trimethoprim (Magiorakos et al., 2012; Thungrat et al., 2015). All MIC determinations were performed in triplicates and *E. coli* ATCC 25922 was used for quality control. The results were interpreted according to the guidelines of Clinical Laboratories Standards Institute (CLSI; CLSI, 2013). The MICs were recorded using the Sensititre Vizion system (Trek Diagnostic Systems), and each isolate was categorized in terms of its resistant phenotype as to: susceptible (S), non-multidrug resistance (DR) or MDR. DR was defined as resistance to 1 or 2 antimicrobial classes, and MDR was defined as resistance to three or more antimicrobial classes.

Additionally, all the 2443 *E. coli* isolates were screened for ESBL production using microdilution-based Sensititre (TREK diagnostic systems, Cleveland, Ohio) with ESBL Confirmatory MIC plates (ESB1F) as described previouly (Aly et al., 2012). Finally, the ESBL-producing isolates expressed MDR phenotype were used in the current study.

### Phylogenetic grouping and virulence genotyping

The distribution of phylogenetic groups amongest the ESBL-producing MDR isolates was determined by the new quadruplex PCR as recently described by Clermont et al. (Clermont et al., 2013). Genomic DNA were extracted from bacterial preparations using the PreMan® Ultra Preparation Reagent according to the manufacturer's protocol. The presence of 17 virulence genes [*fimH, sfa/focDE, afa/draBC, papA, papC, papG alleles* (I, II, III), *hlyA, cnf1, kpsM* II, *fyuA, iutA, ireA, iroN, traT*, and *malX*] known for their association with pathogenicity ExPEC isolates was ascertained in each isolate by use of established PCR assay as reported previously (Johnson and Stell, 2000; Liu et al., 2015).

### Identification of β-lactamase genes and other resistance genes

The occurrence of β-lactamase genes *bla*_CTX−Ms_, *bla*_TEM_, *bla*_SHV_, *bla*_CMY−2_, *bla*_KPC_, *bla*_NDM−1_, and *bla*_OXA−48_ were identified by PCR and subsequent sequencing using specific primers and conditions previously described (Yan et al., 2004; Poirel et al., 2011; Shaheen et al., 2011). Furthermore, the identification of plasmid-mediated quinolone resistance (PMQR) genes [*qnrA, qnrB, qnrC, qnrD, qnrS, aac(6*′*)-Ib-cr* and *qepA*] was carried out as described previously (Liu et al., 2012).

### Transfer of resistance genes by conjugation

We tested whether the ESBL-producing *E. coli* isolates harboring *bla*_CTX−Ms_, *bla*_TEM_, *bla*_SHV_, or *bla*_*OXA*−48_ enzymes were transferable. Conjugation was performed by broth mating at 37°C on 10 ESBL-producing MDR isolates using plasmid-free sodium azide resistant *E. coli* J53 (J53 AZ^r^) as recipient as described previously (Shaheen et al., 2011). Transconjugants were selected on tryptic soy agar plates supplemented with sodium azide (150 μg/ml) and cefotaxime (2 μg/ml). Antimicrobial susceptibility, confirmatory tests for ESBL production, and PCR detection of ESBL genes were performed on all transconjugants as described to confirm transfer of ESBL genes.

### Multilocus sequence typing (MLST)

MLST was performed using seven conserved housekeeping genes of *E. coli* (*adk, fumC, gyrB, icd, mdh, purA*, and *recA*). A detailed scheme of the MLST procedure, including the primers, PCR conditions, allelic type and sequence type assignment methods, is available at MLST databases at the at the Warwick University website (http://mlst.warwick.ac.uk/mlst/dbs/Ecoli).

### Statistical analysis

Significance was determined by Pearson's Chi-squared test with Yates continuity correction using “R” software (version 3.0.1), and the level of significance was set at *P* < 0.05.

## Results

### Antimicrobial susceptibility

Among the 2443 investigated *E. coli* isolates, 92 isolates (3.8%) were ESBL producers, including 68 (73.9%) MDR isolates, 20 (21.5%) DR isolates, and 4 (4.4%) S isolates. Among the 68 ESBL-producing MDR isolates (including 52 dog and 16 cat isolates), 97.1% (66/68) isolates were resistance to cephalothin, followed by cefotaxime (94.1%), ampicillin (92.6%), cefpodoxime (91.2%), amoxicillin-clavulanic acid (86.8%), ticarcillin-clavulanic acid (85.3%), ceftazidime (69.1%), cefoxitin (44.1%), and meropenem (17.6%). Moreover, some of the investigated ESBL-producing MDR isolates were also resistant to non-β-lactam agents, including ciprofloxacin (91.2%), doxycycline (88.2%), enrofloxacin (82.4%), sulfamethoxazole-trimethoprim (50%), chloramphenicol (44.1%), gentamicin (39.7 %), and amikacin (30.9%).

### Phylogenetic groups and the virulence genes distribution

Phylogenetic analysis showed that the predominant phylogenetic groups were D (35.3%) and B2 (33.8%), followed by C (11.8%), A (10.3%), B1 (5.9%), E (1.5%), and F (1.5%). Fourteen of seventeen investigated virulence genes were detected, with the overall occurrence ranging from 11.8% (*ireA*) to 88.2% (*malX*) with the exception of *papG* I and *papG* II, which were not detected in any isolate. The isolates of phylogenic group B2 harbored more virulence genes (mean 7.3), and followed by group B1 (mean 5.8), group D (mean 5.6), group A (mean 4.7), and group C (mean 3.8). Furthermore, CTX-M-producing isolates possessed more virulence genes (mean 8.4) than did non-CTX-M isolates (mean 4.0; *P* < 0.0001). Several virulence genes, including *sfa/focDE, afa/draBC, papA, papC, papG* III, *hlyA, cnf1*, and *iroN*, were significantly more common or even exclusively present in non-CTX-M-producing isolates, whereas *traT* was significantly more common in CTX-M-producing isolates than in non-CTX-M isolates (72 vs. 22.2%, *P* = 0.0008). Additionally, the occurrence of virulence genes among ESBL-producing MDR *E. coli* was significantly lower than among non-ESBL isolates with the exception of *malX* (*P* < 0.01; Table 1).

**Table 1:** **The occurrence of virulence genes in the ESBL-producing and non-ESBL-producing MDR ***E. coli*** isolates**.

	**Occurrence (no. %) of the virulence genes**														
	***malX***	***fimH***	***fyuA***	***traT***	***afa/draBC***	***iutA***	***iroN***	***PapG* III**	***sfa/focDE***	***papC***	***cnf1***	***hlyA***	***papA***	***ireA***	***kpsM* II**
Non-ESBL producers (*n* = 36)	19 (52.8)	30 (83.3)	26 (72.2)	23 (63.9)	32 (88.9)	26 (72.2)	20 (55.6)	11 (30.6)	17 (47.2)	17 (47.2)	18 (50.0)	17 (47.2)	20 (55.6)	7 (19.4)	18 (50.0)
ESBL producers (*n* = 68)	60 (88.2)	47 (69.1)	45 (66.2)	41 (60.3)	39 (57.4)	26 (38.2)	23 (33.8)	16 (23.5)	14 (20.6)	12 (17.6)	11 (16.2)	11 (16.2)	10 (14.7)	7 (10.3)	4 (5.9)
CTX-M producers (*n* = 50)	49 (98)	29 (58.0)	29 (58.0)	36 (72.0)	21 (42.0)	22 (44.0)	9 (18.0)	5 (10)	1 (2.0)	3 (6.0)	2 (4.0)	2 (4.0)	1 (2.0)	1 (2.0)	4 (8)
non-CTX-M producers (*n* = 18)	11 (61.1)	18 (100)	16 (88.9)	5 (27.8)	18 (100)	4 (22.2)	14 (77.8)	11 (61.1)	13 (72.2)	9 (50.0)	9 (50.0)	9 (50.0)	9 (50.0)	6 (33.3)	0 (0)
*P*-value															
ESBL producers vs. nonproducers	0.0012	0.016	0.0166	0.101	0.0005	0.0013	0.0037	0.0096	0.0028	<0.0001	0.0011	0.0017	<0.0001	0.0162	<0.0001
CTX-M producers vs. nonproducers	0.0064	0.0887	0.1056	<0.0001	<0.0001	<0.0001	<0.0001	<0.0001	<0.0001	<0.0001	<0.0001	<0.0001	<0.0001	<0.0001	<0.0001

### Distribution of β-lactamases and PMQR genes

The distribution of β-lactamase and PMQR genes among the 68 ESBL-positive MDR *E. coli* isolates was shown in Table 2. The results showed that *bla*_TEM_, *bla*_SHV_, *bla*_CTX−M_, *bla*_CMY−2_, and *bla*_OXA−48_ were detected in 28 (41.2%), 20 (29.4%), 50 (73.5%), 12 (17.6%), and 13 (19.1%) isolates, respectively. 94.1% (64/68) of the isolates harbored two or more β-lactamase genes, and one isolate from dog with severe urinary tract infection co-harbored eight tested genes [*bla*_*TEM*−5_, *bla*_SHV−12_, *bla*_CMY−2_, *bla*_CTX−M−15_, *bla*_CTX−M−1_, *bla*_CTX−M−14_, *bla*_CTX−M−123_, and *aac(6*′*)-Ib-cr*; Table 2]. For the *bla*_CTX−M_ positive isolates, CTX-M enzymes were clustered in CTX-M-1 (*n* = 35), CTX-M-9 (*n* = 22), and hybrid β-lactamases (*n* = 27) clusters. CTX-M-1 and CTX-M-9 double-positive group accounted for 10.3% of isolates, and three isolates co-harbored CTX-M-1, CTX-M-9 as well as hybrid β-lactamase. *bla*_CTX−M−15_ (*n* = 33) was the predominant genotype in *bla*_CTX−M_ positive isolates, and followed by *bla*_CTX−M−1_ (*n* = 32), *bla*_CTX−M−123_ (*n* = 27), *bla*_CTX−M−9_ (*n* = 19), and *bla*_CTX−M−14_ (*n* = 19). Sequencing of *bla*_TEM_ gene revealed 24 *bla*_TEM−1_, three *bla*_TEM−5_, and one *bla*_TEM−30_, whereas sequencing of *bla*_SHV_ gene revealed 17 *bla*_SHV−12_ and two *bla*_SHV−3_. All *bla*_TEM_ and *bla*_SHV_ genes were classfied as ESBLs with the exception of the *bla*_TEM−1_ gene based on the sequencing. Moreover, 48.5% (33/68) of investigated isolates harbored *aac(6*′*)-Ib-cr*, while none of the isolates carried *qnr* and *qepA* genes. The vast majority of *aac(6*′*)-Ib-cr*-producing isolates were positive for *bla*_CTX−M−15_, *bla*_CTX−M−1_, and *bla*_CTX−M−123_, but negative for CTX-M 9 group enzymes despite *bla*_CTX−M−14_ and *aac(6*′*)-Ib-cr* coexisted in three isolates.

**Table 2:** **Occurrence, diversity, and molecular diversity of ESBL-producing MDR isolates**.

**Sequence types**	**Phylogenetic group**	**Total No. of isolates**	β**-lactamase genes (No. of isolates)**				**PMQR gene**	**Resistance profiles**
			**Non-ESBL**	**ESBL**	**pAmpC**	**Carbapenemase**		
ST5174	F	1		CTX-M-1 + CTX-M-15 + CTX-M-123 (1)	CMY-2 (1)		*aac(6′)-Ib-cr* (1)	AMC, AMP, CIP, CTX, FOX, CPD, CAZ, CEP, CHL, DOX, ENR, GEN, AMK, TIM, SXT
ST1011	E	1		CTX-M-1 + CTX-M-15 (1)			*aac(6′)-Ib-cr* (1)	AMC, AMP, CIP, CTX, FOX, CPD, CAZ, CEP, CHL, DOX, ENR, GEN, AMK, TIM, SXT
ST10	A	1	TEM-1 (1)		CMY-2 (1)			AMC, AMP, CIP, CTX, FOX, CPD, CAZ, CEP, CHL, DOX, ENR, TIM, SXT
ST167	A	2	TEM-1 (1)	CTX-M-1 + CTX-M-15 + CTX-M-123 (2)	CMY-2 (1)		*aac(6′)-Ib-cr* (2)	AMC, AMP, CIP, CTX, FOX, CPD, CAZCHL, DOX, ENR, GEN, TIM, (CEP, AMK)^a^
ST5220	A	1		CTX-M-1 + CTX-M-15 + CTX-M-123 (1)			*aac(6′)-Ib-cr* (1)	AMC, AMP, CIP, CTX, CPD, CEP, DOX, TIM, SXT
ST617	A	1		TEM-5 + CTX-M-15 + CTX-M-123 (1)			*aac(6′)-Ib-cr* (1)	AMC, AMP, CIP, CTX, CPD, CAZ, CEP, DOX, TIM, SXT
ST44	A	1		CTX-M-1 + CTX-M-15 + CTX-M-9 + CTX-M-123 (1)				AMC, AMP, CIP, CTX, CPD, CAZ, CEP, DOX, ENR, TIM, SXT
ST2936	A	1		CTX-M-9 + CTX-M-14 + CTX-M-123 (1)				AMC, CIP, CTX, CPD, CEP, CHL, TIM, SXT
ST2175	B1	1		SHV-12 (1)				FOX, CEP, CHL, DOX, GEN, AMK, SXT
ST443	B1	1		CTX-M-1 + CTX-M-15 (1)			*aac(6′)-Ib-cr* (1)	AMC, AMP, CIP, CTX, CPD, CAZ, CEP, DOX, ENR, TIM
ST162	B1	1		TEM-30 + CTX-M-9 + CTX-M-14 + CTX-M-123 (1)				AMC, CIP, CTX, CPD, CHL, DOX, ENR, TIM, SXT
ST1800	B1	1			CMY-2 (1)	OXA-48 (1)		AMC, AMP, CIP, CTX, FOX, CPD, CAZ, MEM, DOX, ENR, TIM
ST5231	C	1		CTX-M-9+ CTX-M-14 (1)				AMC, AMP, CIP, CTX, CPD, CEP, DOX, ENR, TIM
ST5206	C	1		CTX-M-1+ CTX-M-15+ CTX-M-123 (1)			*aac(6′)-Ib-cr* (1)	AMC, AMP, CIP, CTX, CPD, CAZ, CEP, DOX, ENR, TIM
ST23	C	2	TEM-1 (1)	CTX-M-1 + CTX-M-15 + CTX-M-123 (1)	CMY-2 (1)		*aac(6′)-Ib-cr* (2)	AMC, AMP, CIP, CTX, CPD, CAZ, CEP, DOX, ENR, TIM, (FOX)
ST5232	C	1	TEM-1 (1)				*aac(6′)-Ib-cr* (1)	AMC, AMP, CIP, DOX, ENR, TIM
ST410	C	2	TEM-1 (2)	CTX-M-1 + CTX-M-15 (2), CTX-M-14 (1), CTX-M-123 (1)			*aac(6′)-Ib-cr* (2)	AMC, AMP, CIP, CTX, CPD, CAZ, CEP, DOX, ENR, TIM, SXT
ST1088	C	1	TEM-1 (1)	SHV-3 + CTX-M-1 + CTX-M-15 + CTX-M-123 (1)		OXA-48 (1)	*aac(6′)-Ib-cr* (1)	AMC, AMP, CIP, CTX, CPD, CAZ, CEP, MEM, GEN, AMK, TIM, SXT
ST1722	D	1	TEM-1 (1)		CMY-2 (1)			AMC, AMP, CTX, FOX, CPD, CAZ, CEP, CHL, DOX, TIM, SXT
ST68	D	1		CTX-M-1 + CTX-M-15 + CTX-M-123 (1)			*aac(6′)-Ib-cr* (1)	AMC, AMP, CIP, CTX, FOX, CPD, CAZ, CEP, CHL, DOX, ENR, TIM, SXT
ST69	D	1		CTX-M-1+ CTX-M-15 (1)				AMC, AMP, CPD, CAZ, CEP, DOX, TIM, SXT
ST38	D	3	TEM-1 (1)	TEM-5 + CTX-M-9 + CTX-M-14 + CTX-M-123 (2), SHV-12 (1), CTX-M-1 (1), CTX-M-15 (1),	CMY-2 (2)		*aac(6′)-Ib-cr* (1)	AMC, AMP, CIP, CTX, FOX, CPD, CEP, ENR, DOX, TIM, (GEN, AMK, SXT)
ST405	D	9	TEM-1 (5)	CTX-M-9 + CTX-M-14 (7), CTX-M-1 + CTX-M-15 (2)	CMY-2 (3)	OXA-48 (3)	*aac(6′)-Ib-cr* (2)	AMC, AMP, CIP, CTX, FOX, CPD, CAZ, CEP, CHL, DOX, GEN, TIM, (AMK, MEM, SXT)
ST648	D	9	TEM-1 (2)	SHV-12 (2), CTX-M-1 + CTX-M-15 + CTX-M-123 (3), CTX-M-1 + CTX-M-15 + CTX-M-9 (2), CTX-M-1 + CTX-M-15 (3), CTX-M-9 + CTX-M-14 (1),	CMY-2 (1)	OXA-48 (5)	*aac(6′)-Ib-cr* (6)	AMC, AMP, CIP, CTX, FOX, CPD, CAZ, CEP, DOX, ENR, AMK, TIM, SXT, (CHL, MEM, GEN)
ST131	B2	10	TEM-1 (4)	SHV-3 (1), CTX-M-1 + CTX-M-15 + CTX-M-123 (7), SHV-12 (5), CTX-M-1+ CTX-M-9 + CTX-M-14 (2)		OXA-48 (2)	*aac(6′)-Ib-cr* (7)	AMC, AMP, CTX, CPD, CAZ, CEP, CHL, DOX, ENR, GEN, TIM, (CIP, AMK, SXT)
ST12	B2	2		CTX-M-9 (1), CTX-M-14 (1)		OXA-48 (1)		AMC, AMP, CIP, CTX, CPD, CAZ, CEP, (CHL MEM, DOX, ENR, TIM)
ST5219	B2	1		SHV-12 + CTX-M-14 (1)			*aac(6′)-Ib-cr* (1)	AMP, CTX, CEP, CHL, DOX, ENR
ST961	B2	2		SHV-12 (1), CTX-M-14 (1)			*aac(6′)-Ib-cr* (1)	AMC, CTX, FOX, CEP, CHL, GEN, (CIP, SXT)
ST127	B2	2	TEM-1 (1)	SHV-12 (2)				AMC, AMP, CIP, CTX, FOX, CPD, CEP, CHL, DOX, ENR, (GEN, AMK, TIM, SXT)
ST73	B2	3	TEM-1 (2)	SHV-12 (1)				AMC, AMP, CIP, CTX, CPD, CAZ, CEP, DOX, ENR, TIM
ST372	B2	3		SHV-12 (3)				AMC, AMP, CIP, CTX, FOX, CPD, CEP, CHL, DOX, ENR, (TIM)

*AMC, amoxicillin-clavulanic acid; AMP, ampicillin; CTX, cefotaxime; CAZ, ceftazidime; FOX, cefoxitin; CPD, cefpodoxime; CEP, cephalothin; MEM, meropenem; DOX, doxycycline; CHL, chloramphenicol; CIP, ciprofloxacin; ENR, enrofloxacin; GEN, gentamicin; AMK, amikacin; TIM, ticarcillin-clavulanic acid; SXT, sulfamethoxazole-trimethoprim*.

a *The antibiotics in parentheses indicated that the antibiotics were variability among the isolates*.

### Conjugation experiments

We tested whether *bla*_*CTX*−*M*_ genes or other β-lactamase genes in 10 selected isolates were transferable by conjugation experiments, and seven out of the 10 ESBL-producing isolates successfully transferred the β-lactamase genes to the recipient *E. coli*. PCR analysis showed the presence of respective *bla*_*CTX*−*M*_ genes and other β-lactamase genes, including two *bla*_*OXA*−48_-carrying plasmids from all the transconjugants (Table 3). Meanwhile, PMQR gene *aac-(6')-Ib-cr* was co-transferred with β-lactamase genes. Generally, all donors and their transconjugants were resistant to amoxicillin-clavulanic acid, ampicillin, cefotaxime, cefoxitin, cefpodoxime, cephalothin, and ticarcillin-clavulanic acid, and all transconjugants exhibited an increase of at least eight-fold in MICs compared to the recipient, *E. coli* J53 AZ^r^. The ciprofloxacin MICs for four transconjugants harboring *aac-(6*′*)-Ib-cr* ranged from 0.06 to 0.125 mg/L, representing an increase of two-fold to four-fold compared with the recipient (Table 3). Additionally, the transconjugants remained susceptible to meropenem, ciprofloxacin, gentamicin, chloramphenicol, and doxycycline, whereas one transconjugant harboring *bla*_*OXA*−48_ was resistant to sulfamethoxazole-trimethoprim and reduced the susceptibility to meropenem.

**Table 3:** **Antibimicrobial susceptibility testing profiles of ***E. coli*** isolates used in the conjugation experiments**.

**Strain ID**		**MIC (**μ**g/ml) of antimicrobial agents**															**Presence or absence of**					
		**AMC**	**AMP**	**CTX**	**FOX**	**CPD**	**CAZ**	**CEP**	**TIM**	**MEM**	**CIP**	**GEN**	**CHL**	**DOX**	**SXT**	**TEM**	**SHV**	**CTX-M-15**	**CTX-M-14**	**CTX-M-123**	**OXA-48**	***aac(6′)-Ib-cr***
J53 AZ^*r*^	Recipient	1	4	0.125	0.5	2	0.06	8	2	0.015	0.03	0.25	0.125	0.06	0.06	−	−	−	−	−	−	−
S0544451	Donor	16	512	128	4	256	32	1024	256	0.03	32	32	0.25	1	32	+	+	+	−	+	+	+
Trans-S0544451	Transconjugant	**8**	**512**	**64**	**4**	**64**	**32**	**1024**	**128**	0.015	0.125	0.25	0.125	0.5	0.25	+	−	+	−	+	+	+
H0477803	Donor	32	512	64	32	256	8	1024	64	0.03	16	1	0.25	0.5	1	+	+	+	+	+	−	+
Trans-H0477803	Transconjugant	**32**	**512**	**64**	**16**	**128**	**4**	**1024**	**64**	0.015	0.06	0.125	0.125	0.25	0.25	+	−	+	−	+	−	+
L0812551	Donor	32	512	64	4	256	0.5	1024	256	0.03	64	16	16	32	32	+	−	−	+	+	−	−
Trans-L0812551	Transconjugant	**32**	**512**	**64**	**4**	**256**	**0.5**	**1024**	**256**	0.03	0.015	0.25	0.125	1	**0.5**	−	−	−	+	+	−	−
I0447331	Donor	16	512	128	128	512	16	>1024	128	0.5	64	32	16	32	32	−	+	+	−	−	−	−
Trans- I0447331	Transconjugant	**16**	**512**	**128**	**64**	**512**	**8**	**1024**	**64**	0.015	0.03	0.125	0.125	0.5	**1**	−	+	+	−	−	−	−
I4097858	Donor	16	512	>1024	16	512	32	>1024	512	0.5	32	1	16	32	16	+	−	+	−	−	+	−
Trans-I4097858	Transconjugant	**8**	**256**	**512**	**16**	**256**	**32**	>**1024**	**512**	0.06	0.015	0.25	0.125	0.25	**32**	+	−	+	−	−	+	−
B6276651	Donor	16	512	>1024	8	256	128	>1024	>1024	0.25	128	128	32	16	2	+	+	+	−	+	−	+
Trans-B6276651	Transconjugant	**16**	**512**	>**1024**	**8**	**256**	**64**	**1024**	>**1024**	0.03	0.125	0.25	0.125	0.5	0.06	+	−	−	−	+	−	+
Y0769764	Donor	16	512	128	16	256	64	>1024	256	0.03	32	32	16	16	1	+	−	+	−	+	−	+
Trans-Y0769764	Transconjugant	**16**	**512**	**64**	**8**	**128**	**64**	>**1024**	**256**	0.015	0.125	0.125	0.125	0.5	0.25	−	−	+	−	−	−	+

*Bold values mean that MIC of transconjugant increased at least eight-fold relative to those for the recipient*.

### MLST

The MLST investigation revealed that the 68 isolates were assigned to 31 STs, including six new STs (Table 2). Twelve STs were represented by more than two isolates, and other 19 STs contained a single isolate each. ST131 (*n* = 10), ST648 (*n* = 9), and ST405 (*n* = 9) accounted for more than 41% (28/68) of investigated isolates and 54% (27/50) of CTX-M-producing isolates, respectively. 74.2% (23/31) of STs, especially ST131, ST648, and ST405 were positively associated with CTX-M-producing isolates, while other STs, including ST10, ST5232, ST1722, ST2175, ST1800, ST73, ST372, and ST127 seem to have no relationship with CTX-Ms. Vast majority of ST131 and ST648 isolates were positively associated with *bla*_*CTX*−*M*−15_ and/or *bla*_*CTX*−*M*−1_ as well as *bla*_*CTX*−*M*−123_, whereas 77.8% of ST405 isolates were negatively associated with *bla*_*CTX*−*M*−1_, *bla*_*CTX*−*M*−15_, and *bla*_*CTX*−*M*−123_ genes. Moreover, 55.6% of ST648 isolates were positively associated with *bla*_*OXA*−48_, and 12 pAmpC genes *bla*_*CMY*−2_ were distributed in nine STs. Notably, all ST131, ST405, and ST648 isolates expressed resistance to ciprofloxacin and 3rd generation cephalosporins, whereas all ST131 isolates remained susceptible to cefoxitin. A strong correlation was revealed between the virulence gene profiles and STs, and the same STs showed the similar virulence gene profiles. Among the three most common STs, ST405 isolates harbored more virulence genes (mean 4.6), followed by ST131 (mean 4.4), and virulence genes were less abundant in ST648 isolates (mean 3.4). Almost all of the ST131 and ST405 isolates were positive for *afa/draBC, traT*, and *malX* genes, ST648 isolates were significantly associated with *fimH, malX* and *traT*, but negative for *afa/draBC*.

## Discussion

ESBLs, pAmpC and carbapenemases are mostly responsible for the emerging resistance to the β-lactam antibiotics, especially the 3rd generation cephalosporins and carbapenems in *E. coli* (Pitout, 2012). In the present study, we conducted a molecular detection and characterization of the β-lactamase genes in ESBL-producing MDR *E. coli* isolates from dogs and cats in the United States over a period of time ranging from August 2009 to January 2013, and also revealed the association between the phylogenetic groups, virulence gene profiles, genetic backbones and β-lactamase types.

The prevalence of 3.8% ESBL-producing *E. coli* found in this study is similar to that recorded in a recent study (3%; Shaheen et al., 2011) but higher than the first survey (1%; O'Keefe et al., 2010) among *E. coli* from dogs and cats in the United States. Surprisingly, 73.9% (68/92) of the ESBL-producing *E. coli* exhibited MDR phenotype, and 75% of MDR isolates were resistant to more than 10 antimicrobial agents tested. Phylogenetic groups D and B2 were the main phylogenetic groups in this study, and it was similar to the phylogenetic subtype distribution of the ESBL-producing isolates from human patients (Hu et al., 2013), which further demonstrated that isolates in phylogenetic groups D and B2 were associated with extraintestinal infections. Among the 68 ESBL-producing MDR isolates, *bla*_CTX−M_ was prominent and detected in 73.5% (50/68) of isolates, whereas two previous similar surveys carried out in different states in the United States showed that the corresponding prevalence of *bla*_CTX−M_ were 16.7% and 89.7%, respectively (O'Keefe et al., 2010; Shaheen et al., 2011). It is indicated that the geographical regions, time, resistant phenotype and the history of antimicrobial treatment of the animals can affect the prevalence of *bla*_CTX−M_ gene. The high prevalence of *bla*_CTX−M_ strongly suggests a significant role for *E. coli* isolates from companion animals as ESBL gene reservoirs, which poses an additional risk to humans. Therefore, monitoring of the spread of *bla*_CTX−M_ genes in *E. coli* isolates in dogs and cats is urgently needed. Althouth *bla*_CTX−M−15_ was still the most frequently encountered gene, the specific genotype of *bla*_CTX−M_ is undergoing changes, which was supported by available evidence from the occurrence of CTX-M-9 group as well as the occurrence of a novel hybrid β-lactamase gene *bla*_CTX−M−123_. *bla*_CTX−M−123_ was firstly discovered in *E. coli* from pig feces in China in 2013 (He et al., 2013), and afterward in human specimen (Hu et al., 2013). It is interesting to note that *bla*_CTX−M−15_ is also the most widely distributed ESBL gene among human-associated *Enterobacteriaceae* (Cantón and Coque, 2006). These finding revealed the possibility of cross-transmission between animals and humans. Moreover, several isolates appear only with *bla*_TEM−1_, *bla*_CMY−2_, or *bla*_OXA−48_, suggesting that these isolates perhaps carry other ESBL genes, which will require further studies.

*bla*_CMY−2_ was the most prevalent pAmpC, and it not only confer resistance to a wide range of extended-spectrum cephalosporins but also are not affected by β-lactamase inhibitors. *bla*_CMY−2_ was dected in 17.6% of the isolates in our study, and it was significantly lower than the occurance of *bla*_CMY−2_ (89%) in *E. coli* from companion animals in a previous study in the United States (Shaheen et al., 2011). We supposed that the occurance of *bla*_CMY−2_ might be underestimated since only the ESBL-producing MDR isolates were characterized in this study. Meanwhile, our results showed that majority (58.3%) of CMY-2-producing isolates belonged to phylogenetic group D, consistent with a previous study in *E. coli* from human in Australia (Sidjabat et al., 2014). This similar distribution of phylogenetic group further certified that *bla*_CMY−2_ can also be transferred between different bacterial species and between animals and humans (Li et al., 2007; Shaheen et al., 2011). *bla*_OXA−48_ was initially reported in *Klebsiella pneumoniae* isolates in Turkey in 2001(Poirel et al., 2004) and afterward in other Mediterranean countries (Spain, France, Italy, Egypt, and Lebanon Turkey) (Girlich et al., 2014). In 2013, it was firstly discovered in *E. coli* from dogs in Germany (Stolle et al., 2013). *bla*_OXA−48_ can hydrolyze carbapenems and β-lactamase inhibitors but has no activity toward broad-spectrum cephalosporins (Mathers et al., 2013). Our data showed that about 19% of the isolates carried the *bla*_OXA−48_, and they were mostly associated with meropenem resistance, sequence types ST648, ST405, and ST131 as well as different combinations of β-lactamase genes. To our knowledge, *bla*_OXA−48_ was firstly reported in the United States in 2012 (Poirel et al., 2012), and the present study is the first report of *bla*_OXA−48_ in *E. coli* from dogs and cats in the United States. Moreover, *bla*_OXA−48_ can transfer with other β-lactamases and *aac(6*′*)-Ib-cr*. This finding also revealed possibility of the transfer between humans and companion animals appears highly probable through multiple potential pathways although *bla*_OXA−48_ is still sporadic occurrence in animals.

*aac(6*′*)-Ib-cr* was the exclusive PMQR gene in this study, and CTX-M-producing isolates (particularly *bla*_CTX−M−15_ positive isolates) showed significantly higher occurrence of *aac(6*′*)-Ib-cr* compared to non-CTX-M or non-ESBL isolates (62 vs. 11.1 vs. 10%, *P* < 0.001). The frequent combination of *bla*_CTX−M−15_ and *aac(6*′*)-Ib-cr* in this study further supported the previous studies that coproduction of β-lactamases and PMQR genes could conduce to the dissemination of MDR isolates, and also reflect the fact that genes encoding resistance to β-lactams and quinolones are located on the same plasmid. Although it was not the primary focus of this study, our results coincided with a previous study (Qin et al., 2013) that ESBL-producing isolates presented a lower occurrence of studied virulence genes compared with non-ESBL isolates (the data from another study in our laboratory) with the exception of *malX* gene, and CTX-M-producing *E. coli* harbored fewer virulence genes than non-CTX-M isolates (*P* < 0.0001). A possible reason why individual virulence gene increased among ESBL-producing is that might be a fitness trade-off for the ESBL to survive antibiotics exposure (Qin et al., 2013) and the difference source of *E. coli*. The exact explanation needs additional study in the future.

A previous review suggested that attention should be paid to the rising of *E. coli* ST131, ST648, ST405, and ST38 isolates as they can play an important role in the worldwide distribution of CTX-M-producing *E. coli* (Pitout, 2012). It was further confirmed by our results since ST131, ST648, and ST405 accounted for 54% of the CTX-M-producing MDR isolates. ST131 was the predominant clone in this study, and all ST131 isolates remained susceptible to cefoxitin, which has been recently suggested as an alternative carbapenems for the treatment of infections by ESBL-producing *E. coli* (Guet-Revillet et al., 2014). It is noteworthy that nine ST648 isolates were strongly associated with *bla*_CTX−M−15_ (88.9%, 8/9), *bla*_OXA−48_ (55.6%, 5/9), and severe clinical signs. The zoonotic potential of ST648 ESBL-producing isolates has been indicated in the isolates from humans, domestic and wild animals in previous studies (Nicolas-Chanoine et al., 2008; Cortes et al., 2010), and two recent studies in Europe further suggested that ST648 clone may represent a novel genotype that combines MDR phenotype, extraintestinal virulence and zoonotic potential in companion animals (Huber et al., 2013; Ewers et al., 2014). Furthermore, ST131, ST648, and ST405 isolates have the similar β-lactamase gene combinations and resistance profiles, respectively. While it is alarming that other STs have various β-lactamase gene combinations, especially one ST38 isolate, which was associated with the highest frequency of β-lactamases and *aac(6*′*)-Ib-cr*, high level cephalosporins resistance (MICs ≥ 32 μg/ml), lowest frequency of virulence genes and severe clinical signs. Nevertheless, constant attention and further investigations for ST648 and ST38 isolates in companion animals are necessary as they are now rapidly and globally disseminated as well as the companion animals are more and more considered an important source of human infections as the physical closeness.

## Conclusion

CTX-M-producing *E. coli* tend to have less virulent properties compared with the non-CTX-M isolates. CTX-Ms represented by *bla*_CTX−M−1_, *bla*_CTX−M−15_, and *bla*_CTX−M−123_ have spread rapidly. The occurrence of *bla*_CTX−M−123_ of ESBLs and *bla*_OXA−48_ carbapenemase were particularly striking, being reported here for the first time in *E. coli* from dogs and cats in the United States. ST131, ST648, and ST405 were the predominant clonal groups among the ESBL-producing *E. coli*, and all ST131 isolates remained susceptible to the cefoxitin. This information will be useful for assessing the epidemiological risk factors and appropriate use of antimicrobials for ESBL-producing *E. coli* infections of dogs and cats.

## Author contributions

All authors listed, have made substantial, direct and intellectual contribution to the work, and approved it for publication.

### Conflict of interest statement

The authors declare that the research was conducted in the absence of any commercial or financial relationships that could be construed as a potential conflict of interest.
